# Association of Education and Smoking Status on Risk of Diabetes Mellitus: A Population-Based Nationwide Cross-Sectional Study

**DOI:** 10.3390/ijerph14060655

**Published:** 2017-06-19

**Authors:** Jin-Hyeong Kim, Juhwan Noh, Jae-Woo Choi, Eun-Cheol Park

**Affiliations:** 1 Department of Public Health, Graduate School, Yonsei University, Seoul 03722, Korea; jh.kim910@gmail.com; 2 Department of Preventive Medicine, Yonsei University College of Medicine, Seoul 03722, Korea; njuhwan@yuhs.ac; 3 Busan Public Health Policy Institute, Busan 47527, Korea; jwchoi2695@hanmail.net; 4 Institute of Health Services Research, Yonsei University College of Medicine, Seoul 03722, Korea

**Keywords:** diabetes, education, environmental tobacco smoke, smoker

## Abstract

*Background:* Exposure to smoke, including environmental tobacco smoke (ETS), is a well-known risk factor for diabetes. Low socioeconomic status, especially lack of education, is also a risk factor for diabetes. Therefore, we assessed the association of demographic, socioeconomic, clinical, and behavior risk factor-related variables and smoking status, including ETS exposure, with the prevalence of diabetes. *Methods:* Data were from the 2007–2013 Korea National Health and Nutritional Evaluation Survey (KNHANES). Multivariable logistic regression examined associations between various lifestyle and health factors and the prevalence of diabetes while controlling for potential confounding variables. Subgroup analysis was performed according to smoking status to determine factors associated with diabetes. *Results:* Of 19,303 individuals analyzed, 1325 (11.4%) had diabetes. Greater average age, male sex, lower educational level, unemployment, and coexisting health problems were significantly associated with diabetes. Individuals with only elementary, middle, or high school level education had significantly greater odds ratios (*p* < 0.05) compared to college graduates; smokers and nonsmokers exposed to ETS had significantly greater OR (*p* < 0.05) than nonsmokers unexposed to ETS. Subgroup analysis of diabetics according to smoking status revealed significant associations (*p* < 0.05) for diabetic nonsmokers exposed to ETS with female sex, single status, elementary level education, urban residence, National Health Insurance (NHI), hypertension, a lack of alcohol intake, and a lack of moderate physical activity. For diabetic smokers, there were significant associations (*p* < 0.05) with elementary education, urban residence, a lack of moderate physical activity, a lack of alcohol intake, and NHI. *Conclusions:* The results suggested that smoking status, as well as ETS exposure, was associated with a higher prevalence of diabetes, especially in populations with less education. Thus, we should direct efforts for controlling diabetes toward individuals with lower levels of education and those who are smokers and nonsmokers exposed to ETS.

## 1. Introduction

Smoking is the most commonly known risk factor for diabetes [1]. In 2010, the world prevalence of diabetes among adults (age 20–79 years) was 6.4%, affecting 285 million adults; it is predicted to increase to 7.7%, and 439 million adults by 2030. Between 2010 and 2030, there will be a 69% increase in the number of adults with diabetes in developing countries and a 20% increase in developed countries [2]. In South Korea, aged 30 years or older, about 4.8 million Koreans (13.7%) had diabetes in 2014. In addition, nearly a quarter of Korean adults had prediabetes [3].

Evidence has shown that there is no safe level of exposure to environmental tobacco smoke (ETS) and that exposure leads to serious and often fatal diseases, including cardiovascular and respiratory diseases, as well as lung and other cancers. Children and newborns may also suffer severe harm as a result of ETS exposure [4]. The World Health Organization has estimated that tobacco kills nearly 6 million people each year with 10% of deaths due to ETS exposure [5,6].

There has been interest in the health effects of ETS, which contains >4000 chemical compounds that partly overlap with compounds inhaled in active smoke [7]. A recent study reported that 40% of children, 33% of male nonsmokers, and 35% of female nonsmokers globally have been exposed to ETS [8]. The association between ETS exposure and a significantly increased risk for diabetes was shown in a recent meta-analysis [9].

While there have been some studies that showed an association between smoking (including ETS) and diabetes, demographic, socioeconomic status, clinical, and behavior-related variables have also been considered [10]. Variables related to socioeconomic status have been associated with mortality in diabetic populations and are useful for identifying risk factors for diabetes [11]. A recent study reported socioeconomic inequalities, including education-related disparities in type 2 diabetes [12]. Education level should be considered when managing diabetic patients [13]. Another study described that the individuals with secondhand smoke exposure had higher education status compared to those without secondhand smoke exposure [14].

The identification of risk factors and prevention strategies for diabetes are critical concerns for public health. In addition to smoking, it is important to analyze the relationship between diabetes and ETS exposure. Therefore, we conducted a nationwide cross-sectional study to assess the association between the prevalence of diabetes, educational level, and smoking status, including ETS exposure.

## 2. Materials and Methods

### 2.1. Data Source and Study Population

For this study, we used data from the 2007–2013 Korea National Health and Nutritional Evaluation Survey (KNHANES). KNHANES are cross-sectional surveys that have been conducted annually since 1998 by the Korea Centers for Disease Control and Prevention (KCDC) to assess the health and nutritional status of the Korean population. A stratified multistage cluster-sampling design was used to obtain a nationally representative sample. This survey was composed of three parts: a Health Interview Survey, a Health Examination, and a Nutrition Survey. The overall response rates were 78.4% in 2007–2009, 80.8% in 2010–2012, and 79.3% in 2013. A total of 58,422 individuals (24,781 in 2007–2009; 25,533 in 2010–2012; 8018 in 2013) completed the survey. Individuals who were under 20 years of age were excluded from our analysis. We finally included 19,303 eligible participants in this study. This study was approved by the Institutional Review Board (IRB) from the KCDC, and all participants provided written informed consent (2007-02CON-04-P, 2008-04EXP-01-C, 2009-01CON-03-2C, 2010-02CON-21-C, 2011-02CON-06-C, 2012-01EXP-01-2C, 2013-07CON-03-4C).

### 2.2. Dependent Variable

The dependent variable in this study was the presence of diabetes using two measurements. The first measurement was that individuals answered whether they had ever been diagnosed with diabetes by a physician. Individuals were asked whether they were taking anti-diabetic drugs or starting insulin therapy. Secondly, fasting plasma glucose (FPG) was measured during the health examination. Diabetes was defined as FPG ≥ 126 mg/dL. Based on these measurements for diabetes, we classified all individuals as either having diabetes or not having diabetes. This is in accordance with the US National Institutes of Health and the Korean Diabetes Association criteria [15].

### 2.3. Independent Variables

Demographic, socioeconomic status, clinical, and behavior-related variables as independent risk factors were selected on the basis of a priori information regarding risk factors [12].

Demographic variables included age, gender, marital status and residential location. Socioeconomic status variables included education level, job status, household income, and health insurance type. Clinical and behavioral risk factors included hypertension, dyslipidemia, monthly alcohol intake, physical activity, and smoking status.

Hypertension was defined as a systolic blood pressure (SBP) ≥140 mmHg, a diastolic blood pressure (DBP) ≥90 mmHg, or a use of anti-hypertension drugs. Dyslipidemia was defined as a fasting total cholesterol ≥240 mg/dL or a use of cholesterol drugs. Monthly alcohol intake was classified into two categories: no intake/intake of less than one occasion per month, and intake on more than one occasion per month over the past year. Moderate physical activity was defined as physical activity for 30 min per session more than five times per week.

Smoking status was categorized as nonsmokers unexposed to ETS, nonsmokers exposed to ETS, and current smoker. Respondents answered the following questions: “Were you exposed to ETS in indoor workplaces over the past 7 days?”, “Are there any current smokers other than yourself at home? If so, were you exposed to ETS in the home over the past 7 days?”, “Were you exposed to ETS in any indoor public place, except for smoking areas, over the past 7 days?” Public places included the school, library, public transit, concert hall, and hotels.

### 2.4. Statistical Analyses

We used a multistage, stratified complex survey design. Sampling variability and selection bias were minimized by weighting; specifically, coverage error according to the number of households and individuals, non-response error, and sampling time frame were adjusted. Characteristics of the study population according to the prevalence of diabetes were compared using a chi-square test, and a *p*-value < 0.05 was considered statistically significant. Multivariable logistic regression analysis was used to calculate the odds ratio (OR) with 95% confidence intervals (CI) to evaluate the association between the prevalence of diabetes and independent variables. In the fully adjusted model, all variables were entered simultaneously. An additional subgroup analysis was carried out for diabetics according to smoking status (ETS-unexposed nonsmoker, ETS-exposed nonsmoker, and smoker) to evaluate independent variables. All statistical analyses were performed using the survey procedure in SAS version 9.4 (SAS Institute Inc., Cary, NC, USA).

## 3. Results

Data from 19,303 individuals in the KNHANES 2007–2013 were analyzed in this study, which included 17,040 (88.3%) non-diabetic individuals and 2263 (11.7%) individuals with diabetes. Table 1 shows the characteristics of non-diabetic and diabetic groups and their associations with other covariates. The number of diabetic males (1291/2263, 57.0%) was greater than females (972/2263, 43.0%). There were statistically significant correlations between individuals with diabetes and lower educational levels (*p* < 0.0001): elementary school, 35.1% (795/2263); middle school, 19.2% (434/2263); high school, 24.2% (547/2263); college, 21.5% (487/2263). Regarding smoking status, diabetes patients were as follows: nonsmoker ETS-unexposed, 28.5% (644/2263); nonsmoker ETS-exposed, 13.0% (294/2263); current smoker, 58.6% (1325/2263). The results of associations between diabetes and covariates showed that most covariates had statistically significant correlations with variables of interest, except for moderate physical activity.

Table 2 shows the results of multivariate logistic regression on the prevalence of diabetes. A higher age, male sex, lower educational status, and unemployment were significantly associated with diabetes; marital status and residential location showed no significant association. Educational status showed a significant association with diabetes; furthermore, the OR increased with less education. The ORs were 1.41 (95% CI: 1.13–1.77, *p* < 0.0029) for elementary school or less, 1.33 (95% CI: 1.08–1.65, *p* < 0.0086) for middle school, and 1.30 (95% CI: 1.09–1.54, *p* < 0.0035) for high school. The household income status and health insurance type were not significantly associated with diabetes in this study. Among health factors, individuals with hypertension and dyslipidemia had a higher prevalence of diabetes than individuals without those comorbidities (OR: 2.00, 95% CI: 1.77–2.27, *p* < 0.0001; OR: 2.29, 95% CI: 2.03–2.58, *p* < 0.0001). According to smoking status, compared to nonsmokers unexposed to ETS, the OR was 1.29 (95% CI: 1.07–1.56, *p* < 0.0073) for nonsmokers exposed to ETS and 1.22 (95% CI: 1.02–1.46, *p* < 0.0333) for smokers.

We also performed subgroup analysis to determine variables affecting the relationship between smoking (including ETS exposure) and diabetes (Table 3). Each covariate was evaluated as a subgroup. Subgroup analysis was adjusted to age and other covariates using multivariate logistic regression. Among educational levels, the proportion of individuals with no more than an elementary school education was statistically significant in nonsmokers exposed to ETS and smokers (OR: 1.40, 95% CI: 1.07–1.84, *p* < 0.0148; OR: 1.45, 95% CI: 1.09–1.93, *p* < 0.0099, respectively). College graduates had a significantly higher OR 1.72 (95% CI: 0.97–3.04, *p* = 0.0620) than ETS non-exposed nonsmokers OR 1.76 (95% CI: 1.07–2.90, *p* = 0.0264). Our study reported significant associations between diabetes and urban residence, health insurance type, alcohol intake, or a lack of moderate physical activity, but we could not find any trend.

## 4. Discussion

The proportion of individuals with diabetes continues to grow in South Korea as well as other countries. Smoking is a well-known risk factor for diabetes. While smoking is known to be harmful to our health, ETS also has harmful effects and leads to chronic diseases including diabetes, cardiovascular disease, respiratory disease, and cancer. Thus, it is necessary to the extent to which smoking status, including ETS, is a risk factor for diabetes. Our results suggested a positive relationship between smoking (and ETS) and diabetes according to educational level. Nonsmokers exposed to ETS can be at increased risk for diabetes.

Factors that were significantly associated with the prevalence of diabetes were those associated with health vulnerabilities, including higher age, the female sex, a lower educational level, unemployment, and hypertension or dyslipidemia. With ETS-unexposed nonsmokers as the reference group, nonsmokers exposed to ETS were significantly associated with diabetes. As expected, current smokers were significantly associated with diabetes.

There are reports of a strong association in active smokers and nonsmokers with ETS exposure and subsequent development of impaired fasting glucose or diabetes. Both smokers and nonsmokers exposed to ETS were associated with an increased risk of diabetes [16,17]. Exposure to ETS and active smoking were positively and independently associated with the risk of diabetes [18]. In another cohort study, exposure to ETS was associated with an increased risk of diabetes after adjusting for confounders [19]. This relationship was strengthened by a higher age and physical inactivity [7].

Even for nonsmokers exposed to ETS, our results showed a significant association between smoking status and diabetes. Thus, we confirmed the association between diabetes and smoking status through covariates that were used for subgroup analysis. In our results, lower educational level was related to an increased prevalence of diabetes. As a result of subgroup analysis of diabetics, there was an increasing significant association between smoking status and educational level from college to elementary school levels. This finding held for nonsmokers exposed to ETS as well.

Among nonsmoking women, ETS is prevalent and remains a public health problem [20]. We noted that women with lower levels of education had a higher probability of exposure to ETS [21].

It has been suggested that ETS poses serious health risks to diabetics and additional public health measures are required to reduce overall exposure [22]. It is more important that the government protects nonsmokers from ETS exposure [23].

This study had several strengths compared to previous research. First, we used nationally representative data, so our study results are generalizable to South Korea citizens. Such data are especially helpful in establishing evidence-based health policies. To our knowledge, this is the first attempt to study the relationship between smoking status that includes ETS and diabetes. Therefore, our findings should be helpful in identifying ways to address these critical issues.

Our study also had some limitations. First, because the study focused on the cross-sectional nature of the KNHANES, there may have been weaknesses in fully explaining the causal relationship between smoking-related ETS and diabetes. The data used in this study could not consider time trend for the questionnaire. However, because the study indicated a correlation between smoking including ETS and diabetes, smoking-related factors could be considered as motivations for not smoking in high-risk group of participants. Second, there was a drawback associated with using the smoking variables surveyed. Surely, we could have used urinary cotinine test results, but that test was not consistently employed in 2005 or at any point in the 2007~2012 period. In addition, because we needed environmental tobacco smoking variables to separate ETS-exposed nonsmokers from ETS-unexposed nonsmokers, we had to use the available smoking and ETS variables.

Nonetheless, our results found that both EST-exposed nonsmokers and smokers were significantly associated with the prevalence of diabetes in the low education level population. Thus, it is necessary to construct health policy and preventative efforts for managing the prevalence of diabetes in these populations.

## 5. Conclusions

Our analysis showed that the prevalence of diabetes was significantly higher in nonsmokers with ETS exposure and current smokers compared to nonsmokers without ETS exposure. ETS exposure was especially associated with a presence of diabetes in populations according to education level. Diabetes condition was associated with smoking as well as ETS exposure in populations with the highest education level.

Thus, we suggest a political approach toward addressing diabetes that considers educational level. It is important for people to receive an education about hazard of ETS exposure. Such as preventive education would help the nation’s management of diabetes. Furthermore, we need to expand the scope of the study to examine the effects of ETS exposure in nonsmokers.

## Figures and Tables

**Table 1:** Characteristics of the study population with diabetes in the development dataset; a comparison of factors between non-diabetic and diabetic individuals.

Classification	Non-Diabetes		Diabetes		Total		*p*
**Age (*n*, Mean Age ± SE)**	17,040	49.1 ± 0.2	2263	59.4 ± 0.3	19,303	50.2 ± 0.2	<0.0001
**Gender**							<0.0001
Women	8667	50.9	972	43.0	9639	49.9	
Men	8373	49.1	1291	57.0	9664	50.1	
**Marital Status**							<0.0001
Married	14,127	82.9	1741	76.9	15,868	82.2	
Single	2092	12.3	482	21.3	2574	13.3	
not available	821	4.8	40	1.8	861	4.5	
**Educational Level**							<0.0001
Elementary school	3384	19.9	795	35.1	4179	21.6	
Middle school	2268	13.3	434	19.2	2702	14.1	
High school	4591	26.9	547	24.2	5138	26.6	
College	6797	39.9	487	21.5	7284	37.7	
**Job Status**							<0.0001
Employed	10,462	61.5	1048	46.3	11,510	59.6	
Unemployed	6578	38.6	1215	53.7	7793	40.4	
**Residential Location**							0.0039
Urban	13,116	77.0	1680	74.2	14,796	76.7	
Rural	3924	23.0	583	25.8	4507	23.3	
**Household Income Status**							<0.0001
1 (Lowest)	3294	19.3	786	34.7	4080	21.1	
2	4380	25.8	591	26.2	4971	25.8	
3	4656	27.3	464	20.5	5120	26.5	
4 (Highest)	4710	27.6	422	18.6	5132	26.6	
**Health Insurance Type**							<0.0001
NHI	16,577	97.3	2124	93.9	18,701	96.9	
Medical Aid	463	2.7	139	6.1	602	3.1	
**Hypertension**							<0.0001
No	11,937	70.1	899	39.7	12,836	66.5	
Yes	5103	29.9	1364	60.3	6467	33.5	
**Dyslipidemia**							<0.0001
No	9554	56.1	702	31.0	10,256	53.1	
Yes	7483	43.9	1561	69.0	9047	46.9	
**Monthly Alcohol Intake**							<0.0001
No	7692	45.1	1218	53.8	8910	46.2	
Yes	9348	43.9	1045	46.2	10,393	53.8	
**Moderate Physical Activity**							0.3847
No	15,494	90.9	2045	90.4	17,539	90.9	
Yes	1546	9.1	218	9.6	1764	9.1	
**Smoking Status**							<0.0001
ETS-unexposed nonsmoker	5347	31.4	644	28.5	5991	31.1	
ETS-exposed nonsmoker	2934	17.2	294	13.0	3228	16.7	
Current smoker	8759	51.4	1325	58.5	10,084	52.2	

ETS: environmental tobacco smoke; NHI: National Health Insurance; SE: standard error.

**Table 2:** Multivariate logistic regression on the prevalence of diabetes.

Classification	OR	95% CI	*p*
**Age (*n*, Mean Age ± SE)**	1.03	1.03–1.04	<0.0001
**Gender**			
Women	1.00		
Men	1.53	1.27–1.84	<0.0001
**Marital Status**			
Married	1.00		
Single	1.08	0.92–1.26	0.3725
not applicable	0.83	0.56–1.21	0.3273
**Educational Level**			
Elementary school	1.41	1.13–1.77	0.0029
Middle school	1.33	1.08–1.65	0.0086
High school	1.30	1.09–1.54	0.0035
College	1.00		
**Job Status**			
Employed	1.00		
Unemployed	1.25	1.09–1.43	0.0016
**Residential Location**			
Urban	1.00		
Rural	1.07	0.92–1.25	0.3581
**Household Income Status**			
1 (Lowest)	1.15	0.96–1.38	0.1426
2	1.09	0.92–1.29	0.3061
3	1.05	0.88–1.25	0.6220
4 (Highest)	1.00		
**Health Insurance Type**			
NHI	1.00		
Medical Aid	1.29	0.97–1.72	0.0776
**Hypertension**			
No	1.00		
Yes	2.00	1.77–2.27	<0.0001
**Dyslipidemia**			
No	1.00		
Yes	2.29	2.03–2.58	<0.0001
**Monthly Alcohol Intake**			
No	1.00		
Yes	0.90	0.80–1.01	0.0776
**Moderate Physical Activity**			
No	1.00		
Yes	1.12	0.92–1.36	0.2727
**Smoking Status**			
ETS-unexposed nonsmoker	1.00		
ETS-exposed nonsmoker	1.29	1.07–1.56	0.0073
Current smoker	1.22	1.02–1.46	0.0333

All variables were adjusted by weighting; NHI: National Health Insurance.

**Table 3:** Odds ratios for diabetes for each subgroup by independent risk factors.

Classification	ETS-Unexposed Nonsmoker	ETS-Exposed Nonsmoker			Current Smoker		
	OR	OR	95% CI	*p*	OR	95% CI	*p*
**Gender**							
Women	1.00	1.37	1.10–1.70	0.0046	1.18	0.92, 1.50	0.1924
Men	1.00	1.21	0.75, 1.97	0.4350	1.26	0.89, 1.80	0.1988
**Marital Status**							
Married	1.00	1.23	0.99, 1.54	0.0628	1.27	1.02, 1.57	0.0342
Single	1.00	1.64	1.13, 2.38	0.0090	1.24	0.88, 1.74	0.2162
not applicable	1.00	0.41	0.07, 2.46	0.3287	0.35	0.10, 1.30	0.1185
**Educational Level**							
Elementary school	1.00	1.40	1.07, 1.84	0.0148	1.45	1.09, 1.93	0.0099
Middle school	1.00	0.83	0.53, 1.29	0.4001	0.72	0.45, 1.14	0.1600
High school	1.00	1.24	0.82, 1.90	0.3124	1.23	0.84, 1.80	0.2963
College	1.00	1.72	0.97, 3.04	0.0620	1.76	1.07, 2.90	0.0264
**Job Status**							
Employed	1.00	1.04	0.77, 1.41	0.7946	1.27	0.93, 1.72	0.1282
Unemployed	1.00	1.73	1.33, 2.24	<0.0001	1.10	0.86, 1.41	0.4607
**Residential Location**							
Urban	1.00	1.39	1.12, 1.71	0.0025	1.28	1.04, 1.57	0.0186
Rural	1.00	0.99	0.65, 1.49	0.9428	1.05	0.73, 1.50	0.7958
**Household Income Status**							
1 (Lowest)	1.00	1.12	0.78, 1.61	0.5469	1.24	0.91, 1.70	0.1788
2	1.00	1.23	0.85, 1.78	0.2769	1.19	0.84, 1.68	0.3227
3	1.00	1.46	0.95, 2.24	0.0837	1.48	1.01, 2.19	0.0465
4 (Highest)	1.00	1.28	0.81, 2.01	0.2869	1.28	0.81, 2.01	0.9448
**Health Insurance Type**							
NHI	1.00	1.29	1.06, 1.57	0.0106	1.26	1.04, 1.52	0.0177
Medical Aid	1.00	1.26	0.53, 2.96	0.5999	0.86	0.45, 1.68	0.6671
**Hypertension**							
No	1.00	1.31	0.97, 1.77	0.0840	1.46	1.12, 1.90	0.0050
Yes	1.00	1.30	1.01, 1.67	0.0417	1.06	0.82, 1.36	0.6656
**Dyslipidemia**							
No	1.00	1.44	1.04, 2.00	0.0274	1.33	0.96, 1.83	0.0874
Yes	1.00	1.22	0.97, 1.55	0.0967	1.18	0.95, 1.47	0.1421
**Monthly Alcohol Intake**							
No	1.00	1.38	1.10, 1.73	0.0052	1.52	1.20, 1.93	0.0006
Yes	1.00	1.14	0.80, 1.62	0.4766	0.94	0.69, 1.28	0.7076
**Moderate Physical Activity**							
No	1.00	1.29	1.06, 1.58	0.0114	1.27	1.05, 1.53	0.0147
Yes	1.00	1.25	0.62, 2.51	0.5322	0.75	0.40, 1.41	0.3781

All variables were adjusted by weighting. All subgroup analyses were adjusted according to age and other subgroup variables; NHI: National Health Insurance.
